# Association between pre-existing cardiovascular disease, mortality and cardiovascular outcomes in hospitalised patients with COVID-19

**DOI:** 10.3389/fcvm.2023.1224886

**Published:** 2023-07-05

**Authors:** Hari P. Sritharan, Kunwardeep S. Bhatia, William van Gaal, Leonard Kritharides, Clara K. Chow, Ravinay Bhindi

**Affiliations:** ^1^ Department of Cardiology, Royal North Shore Hospital, Sydney, NSW, Australia; ^2^ Faculty of Medicine and Health, University of Sydney, Sydney, NSW, Australia; ^3^ Department of Cardiology, Bankstown-Lidcombe Hospital, Sydney, NSW, Australia; ^4^ Department of Cardiology, Northern Hospital, Melbourne, VIC, Australia; ^5^ Faculty of Medicine, University of Melbourne, Melbourne, VIC, Australia; ^6^ Department of Cardiology, Concord Repatriation General Hospital, Sydney, NSW, Australia; ^7^ Westmead Applied Research Centre and Department of Cardiology, Westmead Hospital, Sydney, NSW, Australia

**Keywords:** COVID-19, cardiovascular disease, myocardial injury, mortality, troponin

## Abstract

**Background:**

Pre-existing cardiovascular disease and cardiovascular risk factors are common in patients with COVID-19 and there remain concerns for poorer in-hospital outcomes in this cohort. We aimed to analyse the relationship between pre-existing cardiovascular disease, mortality and cardiovascular outcomes in patients hospitalised with COVID-19 in a prospective, multicentre observational study.

**Method:**

This prospective, multicentre observational study included consecutive patients of age ≥18 in their index hospitalisation with laboratory-proven COVID-19 in Australia. Patients with suspected but not laboratory-proven COVID-19 and patients with no available past medical history were excluded. The primary exposure was pre-existing cardiovascular disease, defined as a composite of coronary artery disease, heart failure or cardiomyopathy, atrial fibrillation or flutter, severe valvular disease, peripheral arterial disease and stroke or transient ischaemic attack. The primary outcome was in-hospital mortality. Secondary outcomes were clinical cardiovascular complications (new onset atrial fibrillation or flutter, high-grade atrioventricular block, sustained ventricular tachycardia, new heart failure or cardiomyopathy, pericarditis, myocarditis or myopericarditis, pulmonary embolism and cardiac arrest) and myocardial injury.

**Results:**

1,567 patients (mean age 60.7 (±20.5) years and 837 (53.4%) male) were included. Overall, 398 (25.4%) patients had pre-existing cardiovascular disease, 176 patients (11.2%) died, 75 (5.7%) had clinical cardiovascular complications and 345 (37.8%) had myocardial injury. Patients with pre-existing cardiovascular disease had significantly increased in-hospital mortality (aOR: 1.76 95% CI: 1.21–2.55, *p* = 0.003) and myocardial injury (aOR: 3.27, 95% CI: 2.23–4.79, *p* < 0.001). There was no significant association between pre-existing cardiovascular disease and in-hospital clinical cardiovascular complications (aOR: 1.10, 95% CI: 0.58–2.09, *p* = 0.766). On mediation analysis, the indirect effect and Sobel test were significant (*p* < 0.001), indicating that the relationship between pre-existing cardiovascular disease and in-hospital mortality was partially mediated by myocardial injury. Apart from age, other cardiovascular risk factors such as diabetes, hypercholesterolemia and hypertension had no significant impact on mortality, clinical cardiovascular complications or myocardial injury.

**Conclusions:**

Pre-existing cardiovascular disease is associated with significantly higher mortality in patients hospitalised with COVID-19. This relationship may be partly explained by increased risk of myocardial injury among patients with pre-existing cardiovascular disease which in turn is a marker associated with higher mortality.

## 1. Introduction

Coronavirus disease 2019 (COVID-19) caused by severe acute respiratory syndrome coronavirus 2 (SARS-CoV-2), was declared a pandemic in March 2020 with over 660 million cases and 6.5 million deaths to date (1). COVID-19 can affect the heart and vascular tissues through ACE2 (angiotensin-converting enzyme 2), the host cell receptor for the viral spike protein of SARS-CoV-2 and has been linked with cardiovascular complications, such as arrhythmias, myocardial injury and heart failure (2, 3). Pre-existing cardiovascular disease and cardiovascular risk factors are common in patients hospitalised with COVID-19, and given the cardiac manifestations of COVID-19 there remain concerns for poorer in-hospital outcomes in this patient cohort (4). These concerns stem in part from the increased mortality and cardiovascular complications in patients with pre-existing cardiovascular disease hospitalised with influenza (5).

Previous studies reporting on the link between pre-existing cardiovascular disease and COVID-19-related outcomes are conflicting, and largely limited by either having a single centre experience, non-generalisable population limited to the critical care setting or reduced data granularity associated with administrative databases (6–10). Moreover, healthcare systems across the world have faced considerable strain due to COVID-19, and the extent this pandemic context confounds the results of studies investigating COVID-19 outcomes, many performed in the earlier phases of the pandemic, remains unknown. Australia provides a unique context to study the outcomes of COVID-19 patients, with its geographical isolation and strict social distancing laws in the earlier phases of the pandemic mediating lower case numbers such that hospital systems could maintain the necessitated high level of care to all patients.

We present an analysis of the relationship between pre-existing cardiovascular disease, mortality and cardiovascular complications through leveraging the Australian Cardiovascular COVID-19 (AUS-COVID) registry, the largest Australian multicentre COVID-19 cardiovascular registry of adult hospitalised patients.

## 2. Materials and methods

### 2.1. Study design

The AUS-COVID study is a prospective, multicentre, observational cohort study across 21 hospitals in Australia. The study was approved by the Northern Sydney Local Health District Human Research Ethics Committee (HREC 2020/ETH00732), who granted a waiver of consent. The registry was prospectively registered with the Australian and New Zealand Clinical Trials Registry (ACTRN12620000486921).

### 2.2. Study population

All index hospitalisations of consecutive adult patients (≥18 years) with laboratory-proven SARS-CoV-2 infection (nucleic acid testing, serology testing or antigen detection) were included. Patients with suspected but not laboratory-proven SARS-CoV-2 infection and patients with no available past medical history were excluded. Patients were also excluded if they were transferred to another hospital, as their mortality and cardiovascular outcomes data would not be available. Patients were excluded if they were transferred from another hospital to avoid recruitment bias, given majority of the included sites were major tertiary centres.

### 2.3. Exposure and outcomes

The primary exposure was pre-existing cardiovascular disease, defined as a composite of a history of coronary artery disease, heart failure or cardiomyopathy, atrial fibrillation or flutter, severe valvular disease, peripheral arterial disease and stroke or transient ischaemic attack (TIA), as documented in the medical records at time of admission. The secondary exposures were age, gender, hypertension, hypercholesterolemia, diabetes mellitus, current or recent smoking status (<1 year) and chronic kidney disease (eGFR < 60 ml/min/1.73 m^2^).

The primary outcome was in-hospital mortality. The secondary outcomes were clinical cardiovascular complications as both individual and composite outcomes during hospitalisation, and myocardial injury during hospitalisation. Clinical cardiovascular complications during hospitalisation were defined as a composite endpoint of new onset atrial fibrillation or flutter, high-grade atrioventricular block, sustained ventricular tachycardia, new heart failure or cardiomyopathy, pericarditis, myocarditis or myopericarditis, pulmonary embolism and cardiac arrest. Myocardial injury was defined as troponin greater than the upper limit of normal for each site-specific assay. Pre-existing coronary artery disease was defined as prior myocardial infarction, percutaneous coronary intervention, coronary artery bypass graft surgery, angina or greater than 50% stenosis of an epicardial vessel on coronary angiography. Heart failure or cardiomyopathy includes patients both with and without reduced left ventricular ejection fraction. Atrial fibrillation was defined as patients with paroxysmal, persistent and permanent atrial fibrillation.

### 2.4. Statistical analysis

Continuous variables were reported as mean (standard deviation) and categorical variables were presented as proportions. Comparisons between groups were made with independent-samples t-test, Pearson's Chi-square test and Fisher's exact test for normally distributed continuous variables, categorical variables with expected cell size ≥5 and categorical variables with expected cell size <5 respectively.

Multivariable binary logistic regression was performed to calculate adjusted odds ratios for mortality, clinical cardiovascular complications during hospitalisation and myocardial injury based on the primary exposure of pre-existing cardiovascular disease. The secondary exposure variables detailed earlier were all considered in the multivariable models. Stepwise models were created with each outcome as the dependent variable; model 0 was unadjusted and model 1 included covariates with *p* value <0.25 on univariate analysis.

Mediation analysis was conducted to examine whether the relationship between pre-existing cardiovascular disease and in-hospital mortality was mediated by myocardial injury. This analysis was conducted using the following steps: first, we regressed in-hospital mortality on pre-existing cardiovascular disease; second, we regressed myocardial injury on pre-existing cardiovascular disease; and third, we regressed pre-existing cardiovascular disease on both in-hospital mortality and myocardial injury. The indirect effect of pre-existing cardiovascular disease on in-hospital mortality through myocardial injury was estimated using the Sobel test.

Statistical analysis was performed using IBM SPSS Statistics Subscription [Version 29.0.0.0 (241)]. Results were considered statistically significant if the 2-sided *p* value was <0.05.

## 3. Results

**Figure 1:**
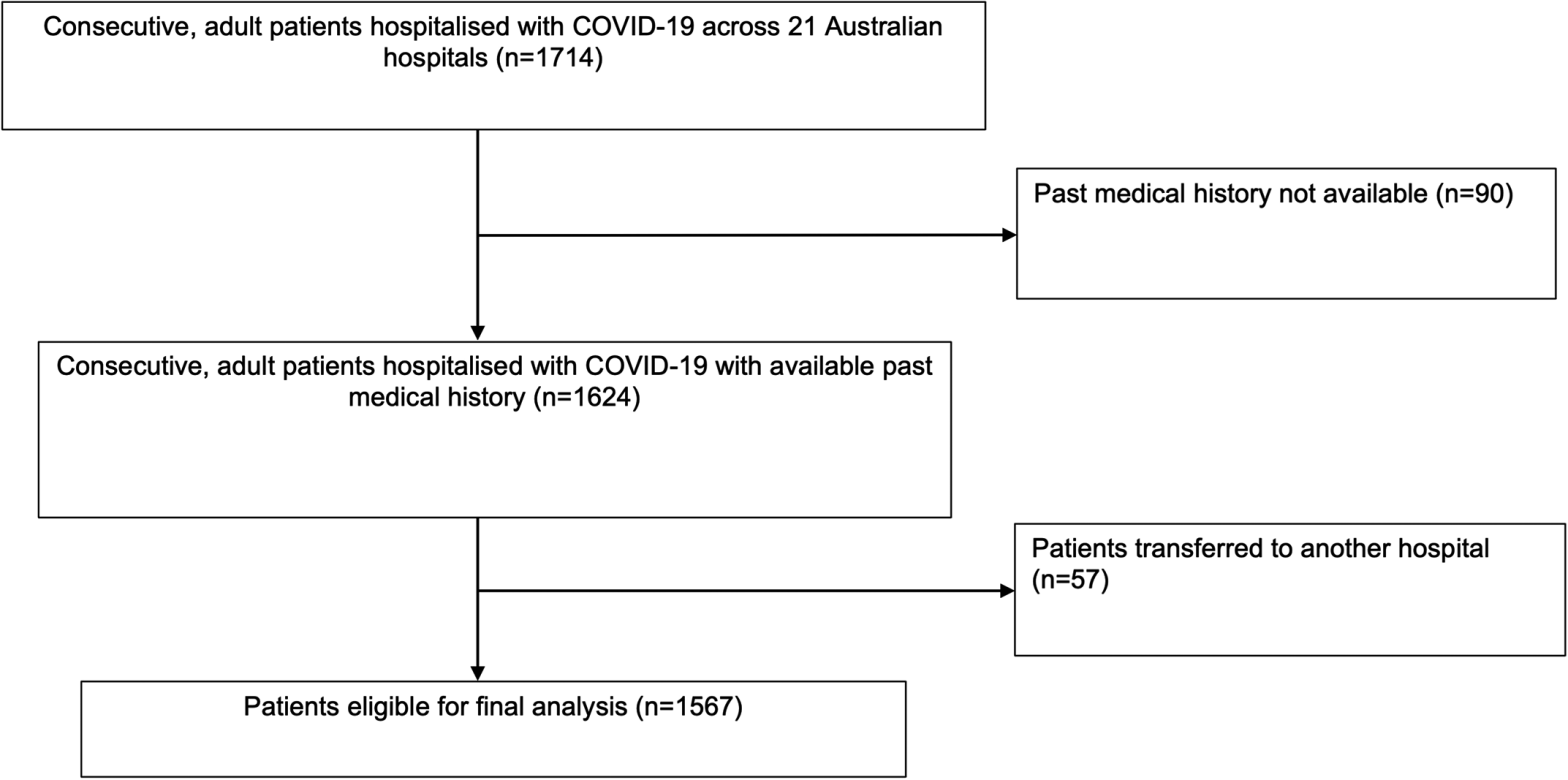
STROBE flow diagram of study population.

1,714 patients were included in the AUS-COVID registry with laboratory-confirmed SARS-CoV-2 infection across 21 Australian hospitals. 1,567 patients were eligible for inclusion in the primary analysis (Figure 1). 90 patients were excluded because their past medical history was unavailable, and 57 patients were excluded because they were transferred to another hospital on discharge. The known admission dates for patients included in this study were from 04/01/2021 to 11/07/2022. This includes patients across time periods where the Alpha (B.1.1.7), Delta (B.1.617.2) and Omicron (B.1.1.529) variants of SARS-CoV-2 predominated in Australia.

Table 1 summarises the demographics, clinical characteristics and the baseline medication exposure of patients on admission. The mean age of included patients was 60.7 (±20.5) years and 837 (53.4%) patients were males. Overall, 398 (25.4%) patients had pre-existing cardiovascular disease. Of these, 174 (11.1%) patients had coronary artery disease, 106 (6.8%) patients had heart failure or cardiomyopathy, 162 (10.3%) patients had atrial fibrillation or flutter, 40 (2.6%) patients had severe valvular disease, 93 (5.9%) patients had stroke or transient ischaemic attack and 21 (1.3%) patients had peripheral arterial disease. Patients with pre-existing cardiovascular disease were older (76.2 ± 14.6 years vs. 55.4 ± 19.6 years, *p* < 0.001) and a significantly higher incidence of hypertension (71.9% vs. 34.2%, *p* < 0.001), hypercholesterolemia (48.2% vs. 21.9%, *p* < 0.001), diabetes mellitus (35.4% vs. 19.8%, *p* < 0.001) and chronic kidney disease (19.1% vs. 4.3%, *p* < 0.001).

**Table 1:** Demographics and clinical characteristics on admission.

	All patients (*n* = 1,567)	Patients without pre-existing cardiovascular disease (*n* = 1,169)	Patients with pre-existing cardiovascular disease (*n* = 398)	*p* value
**Demographics**				
Mean age (SD), yr	60.7 (20.5)	55.4 (19.6)	76.2 (14.6)	<0.001
Male, no. (%)	837 (53.4)	609 (52.1)	228 (57.3)	0.073
Healthcare worker, no. (%)	62 (4.0)	57 (4.9)	5 (1.3)	0.001
Nursing home resident, no. (%)	137 (8.7)	56 (4.8)	81 (20.4)	<0.001
**Cardiovascular risk factors**				
Hypertension, no (%)	686 (43.8)	400 (34.2)	286 (71.9)	<0.001
Hypercholesterolemia, no. (%)	448 (28.6)	256 (21.9)	192 (48.2)	<0.001
Diabetes mellitus, no. (%)	372 (23.7)	231 (19.8)	141 (35.4)	<0.001
Current or recent smoker (<1 year), no. (%)	127 (8.1)	100 (8.6)	27 (6.8)	0.264
Chronic kidney disease (eGFR < 60 ml/min/1.73 m^2^), no. (%)	126 (8.0)	50 (4.3)	76 (19.1)	<0.001
**Cardiovascular disease**				
Coronary artery disease, no. (%)	174 (11.1)	0 (0.0)	174 (43.7)	<0.001
Heart failure or cardiomyopathy, no. (%)	106 (6.8)	0 (0.0)	106 (26.6)	<0.001
Atrial fibrillation or flutter, no. (%)	162 (10.3)	0 (0.0)	162 (40.7)	<0.001
Severe valvular disease, no. (%)	40 (2.6)	0 (0.0)	40 (10.1)	<0.001
Stroke or transient ischemic attack, no. (%)	93 (5.9)	0 (0.0)	93 (23.4)	<0.001
Peripheral arterial disease, no. (%)	21 (1.3)	0 (0.0)	21 (5.3)	<0.001
**Vaccination status** ^a^				
Unvaccinated	199 (25.8)	164 (29.3)	35 (16.7)	<0.001
1 dose, no. (%)	101 (13.1)	76 (13.6)	25 (11.9)	0.529
2 doses, no. (%)	199 (25.8)	125 (22.3)	74 (35.2)	<0.001
3 doses, no. (%)	36 (4.7)	21 (3.8)	15 (7.1)	0.049
Unknown, no. (%)	234 (30.4)	172 (31.1)	61 (29.0)	0.631
**Medications** ^b^				
Aspirin, no. (%)	219 (15.5)	86 (8.3)	133 (35.1)	<0.001
P2Y12 inhibitor, no. (%)	55 (3.9)	8 (0.8)	47 (12.4)	<0.001
Warfarin, no. (%)	18 (1.3)	3 (0.3)	15 (4.0)	<0.001
Novel oral anticoagulants, no. (%)	118 (8.3)	16 (1.5)	102 (26.9)	<0.001
Angiotensin converting enzyme inhibitor, no. (%)	188 (13.3)	105 (10.1)	83 (21.9)	<0.001
Angiotensin receptor blocker, no. (%)	270 (19.1)	181 (17.5)	89 (23.5)	0.011
Mineralocorticoid receptor antagonist, no. (%)	44 (3.1)	10 (1.0)	34 (9.0)	<0.001
Loop diuretic, no. (%)	144 (10.2)	35 (3.4)	109 (28.8)	<0.001
Thiazide diuretic, no. (%)	86 (6.1)	57 (5.5)	29 (7.7)	0.135
Beta blocker, no. (%)	254 (18.0)	69 (6.7)	185 (48.8)	<0.001
Non-dihydropyridine calcium channel blocker, no. (%)	16 (1.1)	7 (0.7)	9 (2.4)	0.007
Dihydropyridine calcium channel blocker, no. (%)	197 (13.9)	130 (12.6)	67 (17.7)	0.014
Nitrates, no. (%)	32 (2.3)	3 (0.3)	29 (7.7)	<0.001

^a^ For patients in each group with described vaccination status.

^b^ For “All patients”, missing data for 153 patients (9.8%). For “Patients without cardiovascular disease”, missing data for 134 patients (11.5%). For “Patients with cardiovascular disease”, missing data for 19 patients (4.8%).

Table 2 details the primary and secondary outcomes and markers of disease severity for included patients. During hospitalisation, 176 patients (11.2%) died, 75 (5.7%) had clinical cardiovascular complications and 345 (37.8%) had myocardial injury. Patients with pre-existing cardiovascular disease had a higher incidence of mortality (25.6% vs. 6.3%, *p* < 0.001), clinical cardiovascular complications (8.5% vs. 5.2%, *p* = 0.078) and myocardial injury (72.5% vs. 25.1%, *p* < 0.001). With respect to markers of COVID-19 disease severity, 254 patients (16.2%) were admitted to the intensive care unit, with a significantly lower proportion of patients with pre-existing cardiovascular disease admitted to ICU compared to those without pre-existing cardiovascular disease (11.6% vs. 17.8%, *p* < 0.004). 116 (7.4%) patients were intubated, 64 (4.1%) patients were given intravenous positive inotropes or vasopressors and 2 (0.1%) patients were placed on extracorporeal membrane oxygenation. Coronary angiography was performed in 9 patients; 1 patient proceeded to have percutaneous coronary intervention and 1 patient had coronary artery bypass graft surgery for triple vessel coronary artery disease.

**Table 2:** Primary and secondary outcomes and markers of COVID-19 disease severity for patients with and without pre-existing cardiovascular disease.

	All patients (*n* = 1,567)	Patients without pre-existing cardiovascular disease (*n* = 1,169)	Patients with pre-existing cardiovascular disease (*n* = 398)	Odds ratio (95% CI)	*p* value
**Primary outcome**					
All-cause, in hospital mortality, no. (%)	176 (11.2)	74 (6.3)	102 (25.6)	5.10 (3.68 to 7.06)	<0.001
Cardiovascular cause of death, no. (%)	12 (0.8)	4 (0.3)	8 (2.0)	5.97 (1.79 to 19.95)	0.004
Other cause of death, no. (%)	164 (10.5)	70 (6.0)	94 (23.6)	4.84 (3.46 to 6.76)	<0.001
**Secondary outcomes**					
Clinical cardiovascular complications, no. (%)	76 (5.7)^a^	61 (5.2)^b^	15 (8.5)^c^	1.69 (0.94 to 3.04)	0.078
New onset atrial fibrillation or flutter, no. (%)	34 (2.4)^d^	26 (2.2)^e^	8 (3.4)^f^	1.54 (0.69 to 3.45)	0.289
High-grade atrioventricular block, no. (%)	5 (0.3)	2 (0.2)	3 (0.8)	4.43 (0.74 to 26.62)	0.107
Sustained conscious ventricular tachycardia, no. (%)	2 (0.1)	1 (0.1)	1 (0.3)	2.94 (0.18 to 47.15)	0.444
New heart failure or cardiomyopathy, no. (%)	13 (0.9)^g^	11 (0.9)^h^	2 (0.7)^i^	0.73 (0.16 to 3.29)	0.676
Pericarditis, no. (%)	2 (0.1)	2 (0.2)	0 (0.0)	1.34 (1.30 to 1.38)	1.000
Myocarditis or myopericarditis, no. (%)	5 (0.3)	5 (0.4)	0 (0.0)	1.34 (1.30 to 1.38)	0.338
Pulmonary embolism, no (%)	24 (1.5)	16 (1.4)	8 (2.0)	1.48 (0.63 to 3.48)	0.369
Cardiac arrest, no. (%)	13 (0.8)	8 (0.7)	5 (1.3)	1.85 (0.60 to 5.68)	0.277
Myocardial injury, no. (%)	345 (37.8)^j^	168 (25.1)^k^	177 (72.5)^l^	7.86 (5.65 to 10.95)	<0.001
**Markers of COVID-19 disease severity**					
ICU admission, no. (%)	254 (16.2)	208 (17.8)	46 (11.6)	0.60 (0.43 to 0.85)	0.004
Intubation, no. (%)	116 (7.4)	95 (8.1)	21 (5.3)	0.63 (0.39 to 1.03)	0.061
Intravenous positive inotropes or vasopressors, no. (%)	64 (4.1)	52 (4.4)	12 (3.0)	0.67 (0.35 to 1.26)	0.212
Extracorporeal membrane oxygenation (ECMO), no. (%)	2 (0.1)	2 (0.2)	0 (0.0)	1.34 (1.30 to 1.38)	1.000

^a^ Out of 1,343 patients without pre-existing atrial fibrillation or flutter and pre-existing heart failure.

^b^ Out of 1,167 patients without pre-existing atrial fibrillation or flutter and pre-existing heart failure.

^c^ Out of 176 patients without pre-existing atrial fibrillation or flutter and pre-existing heart failure.

^d^ Out of 1,404 patients without pre-existing atrial fibrillation or flutter.

^e^ Out of 1,168 patients without pre-existing atrial fibrillation or flutter.

^f^ Out of 236 patients without pre-existing atrial fibrillation or flutter.

^g^ Out of 1,460 patients without pre-existing heart failure.

^h^ Out of 1,168 patients without pre-existing heart failure.

^i^ Out of 292 patients without pre-existing heart failure.

^j^ Out of 912 patients who had troponin measured during admission.

^k^ Out of 668 patients who had troponin measured during admission.

^l^ Out of 244 patients who had troponin measured during admission.

### 3.1. In-hospital mortality

On univariable analysis, pre-existing cardiovascular disease (OR: 5.10, 95% CI: 3.68–7.06, *p* < 0.001), age (OR: 1.09, 95% CI: 1.08–1.11, *p* < 0.001), hypertension (OR: 3.22, 95% CI: 2.30–4.50, *p* < 0.001), hypercholesterolemia (OR: 1.87, 95% CI: 1.35–2.58, *p* < 0.001), diabetes mellitus (OR: 1.84, 95% CI: 1.32–2.58, *p* < 0.001) and chronic kidney disease (OR: 4.08, 95% CI: 2.68–6.20, *p* < 0.001) were associated with significantly higher odds of in-hospital mortality (Table 3). Current or recent smoking status (OR: 0.44, 95% CI: 0.20–0.96, *p* = 0.033) was also associated with significantly decreased odds of in-hospital mortality on univariable analysis.

**Table 3:** Association between pre-existing cardiovascular disease and in-hospital mortality, clinical cardiovascular complications & myocardial injury.

Variable	MODEL 0^a^						MODEL 1^b^					
	Odds Ratio (95% CI) for in-hospital mortality	*p* value	Odds ratio (95% CI) for clinical cardiovascular complications	*p* value	Odds Ratio (95% CI) for myocardial injury	*p* value	Adjusted Odds Ratio (95% CI) for in-hospital mortality	*p* value	Adjusted Odds ratio (95% CI) for clinical cardiovascular complications	*p* value	Adjusted Odds ratio (95% CI) for myocardial injury	*p* value
Pre-existing cardiovascular disease	5.10 (3.68–7.06)	<0.001	1.69 (0.94–3.04)	0.078	7.86 (5.65–10.95)	<0.001	1.76 (1.21–2.55)	0.003	1.10 (0.58–2.09)	0.766	3.27 (2.23–4.79)	<0.001
Age	1.09 (1.08–1.11)	<0.001	1.02 (1.01–1.03)	<0.001	1.07 (1.06–1.08)	<0.001	1.09 (1.07–1.10)	<0.001	1.02 (1.00–1.03)	0.030	1.06 (1.05–1.07)	<0.001
Male	1.17 (0.85–1.60)	0.337	1.27 (0.79–2.02)	0.325	1.26 (0.96–1.66)	0.090	N/A	N/A	N/A	N/A	1.38 (0.98–1.93)	0.063
Hypertension	3.22 (2.30–4.50)	<0.001	1.69 (1.07–2.69)	0.025	3.84 (2.89–5.09)	<0.001	0.99 (0.66–1.48)	0.946	1.07 (0.61–1.87)	0.821	1.19 (0.81–1.74)	0.384
Hypercholesterolemia	1.87 (1.35–2.58)	<0.001	1.71 (1.05–2.76)	0.029	2.36 (1.77–3.15)	<0.001	0.79 (0.54–1.15)	0.221	1.21 (0.70–2.08)	0.504	0.83 (0.57–1.22)	0.354
Diabetes mellitus	1.84 (1.32–2.58)	<0.001	1.47 (0.89–2.44)	0.135	2.00 (1.46–2.73)	<0.001	1.06 (0.73–1.55)	0.752	1.04 (0.60–1.81)	0.885	1.07 (0.72–1.59)	0.729
Current or recent smoker (<1 year)	0.44 (0.20–0.96)	0.033	0.60 (0.21–1.66)	0.318	0.82 (0.51–1.31)	0.409	0.75 (0.33–1.75)	0.508	N/A	N/A	N/A	N/A
Chronic kidney disease (eGFR < 60 ml/min/1.73 m^2^)	4.08 (2.68–6.20)	<0.001	2.30 (1.10–4.80)	0.023	7.80 (4.49–13.53)	<0.001	1.83 (1.14–2.94)	0.013	1.65 (0.76–3.58)	0.209	4.41 (2.29–8.47)	<0.001

^a^ Model 0: Unadjusted.

^b^ Model 1: Variables in Model 0 with *p* < 0.25.

**Figure 2:**
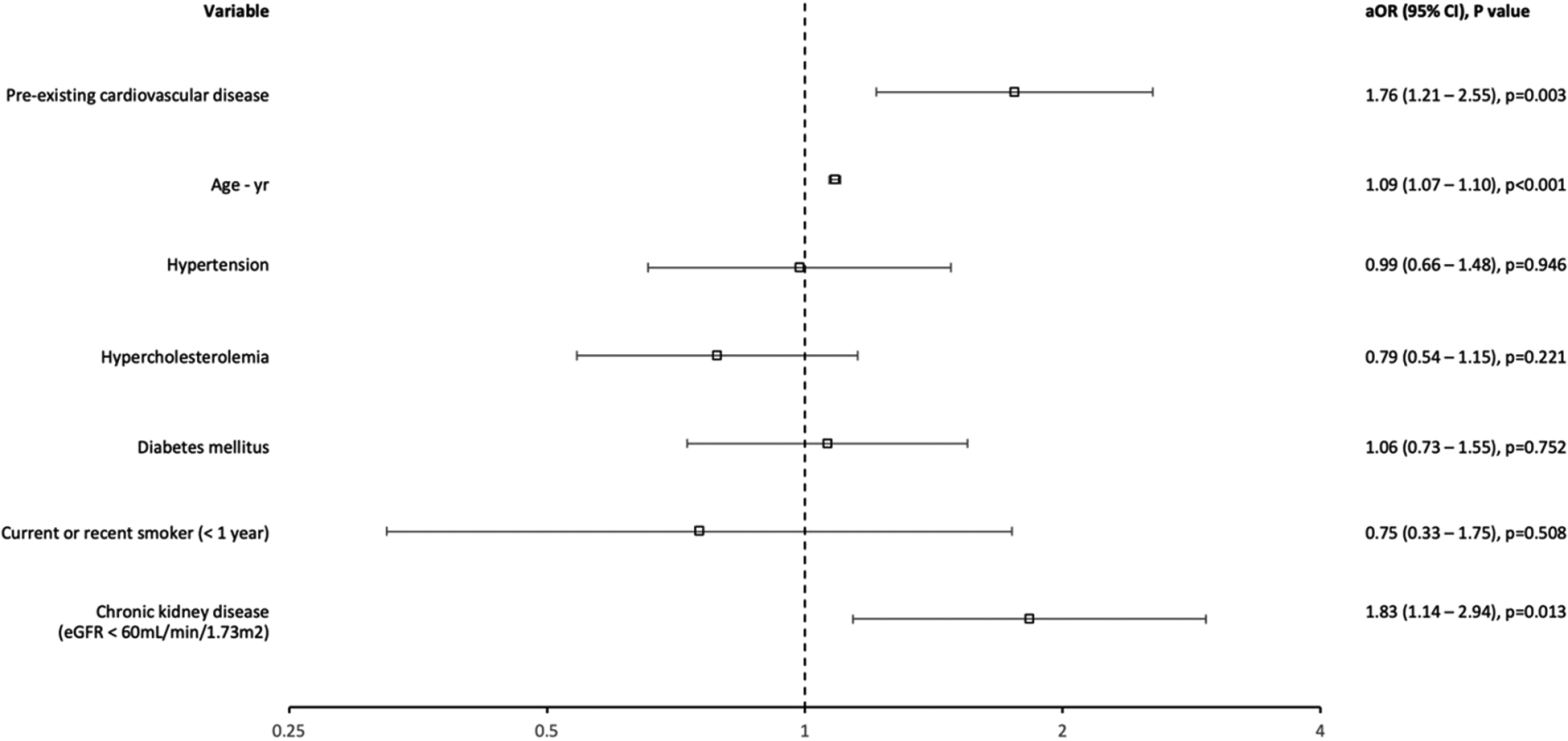
Forrest plot for multivariable logistic regression for in-hospital mortality.

In the multivariable model, pre-existing cardiovascular disease (aOR: 1.76, 95% CI: 1.21–2.55, *p* = 0.003), age (aOR: 1.09, 95% CI: 1.07–1.10, *p* < 0.001) and chronic kidney disease (aOR: 1.83, 95% CI: 1.14–2.94, *p* = 0.013) were associated with significantly increased odds of in-hospital mortality (Figure 2, Table 3). The multivariable binary logistic regression model was statistically significant [*χ*^2^(7) = 274.295, *p* < 0.001], explained 31.8% (Nagelkerke *R*^2^) of the variance in in-hospital mortality and correctly classified 88.6% of cases. The model fit the data well using the Hosmer-Lemeshow goodness-of-fit test [*χ*^2^(8) = 7.897, *p* = 0.444].

### 3.2. Clinical cardiovascular complications

There was no statistically significant association between pre-existing cardiovascular disease and the individual clinical cardiovascular complication endpoints of new onset atrial fibrillation or flutter (OR: 1.54, 95% CI: 0.69–3.45, *p* = 0.289), high-grade atrioventricular block (OR: 4.43, 95% CI: 0.74–26.62, *p* = 0.107), sustained conscious ventricular tachycardia (OR: 2.94, 95% CI: 0.18–47.15, *p* = 0.444), new heart failure or cardiomyopathy (OR: 0.73, 95% CI: 1.30–1.38, *p* = 1.000), myocarditis or myopericarditis (OR: 1.34, 95% CI: 1.30–1.38, *p* = 0.338), pulmonary embolism (OR: 1.48, 95% CI: 0.63–3.48, *p* = 0.369) and cardiac arrest (OR: 1.85, 95% CI: 0.60–5.68, *p* = 0.277) (Table 2).

With respect to the composite endpoint of clinical cardiovascular complications, age (OR: 1.02, 95% CI: 1.01–1.03, *p* < 0.001), hypertension (OR: 1.69, 95% 1.07–2.70, *p* = 0.025), hypercholesterolemia (OR: 1.71, 95% CI: 1.05–2.76, *p* = 0.029) and chronic kidney disease (OR: 2.30, 95% CI: 1.10–4.80, *p* = 0.023) were associated with significantly increased odds on univariable analysis. There was no statistically significant association between pre-existing cardiovascular disease (OR: 1.69, 95% CI: 0.94–3.04, *p* = 0.078) and in-hospital clinical cardiovascular complications on univariable analysis. In the multivariable model, only age (aOR: 1.02, 95% CI: 1.00–1.03, *p* = 0.030) was significantly associated with increased odds of clinical cardiovascular complications; pre-existing cardiovascular disease (aOR: 1.10, 95% CI: 0.58–2.09, *p* = 0.766) had no significant association (Table 3). The multivariable binary logistic regression model was statistically significant [*χ*^2^(6) = 14.225, *p* = 0.027], explained 3.0% (Nagelkerke *R*^2^) of the variance in clinical cardiovascular complications and correctly classified 94.3% of cases. The model fit the data well using the Hosmer-Lemeshow goodness-of-fit test [*χ*^2^(8) = 9.973, *p* = 0.267].

### 3.3. Myocardial injury

On univariable analysis, pre-existing cardiovascular disease (OR: 7.86, 95% CI: 5.65–10.95, *p* < 0.001), age (OR: 1.02, 95% CI: 1.01–1.03, *p* < 0.001), hypertension (OR: 3.84, 95% CI: 2.89–5.09, *p* < 0.001), hypercholesterolemia (OR: 2.36, 95% CI: 1.77–3.15, *p* < 0.001), diabetes mellitus (OR: 2.00, 95% CI: 1.46–2.73, *p* < 0.001) and chronic kidney disease (OR: 7.80, 95% CI: 4.49–13.53, *p* < 0.001) were associated with significantly increased odds of myocardial injury.

In the multivariable model, pre-existing cardiovascular disease (aOR: 3.27, 95% CI: 2.23–4.79, *p* < 0.001), age (aOR: 1.06, 95% CI: 1.05–1.07, *p* < 0.001) and chronic kidney disease (aOR: 4.41, 95% CI: 2.29–8.47, *p* < 0.001) were associated with significantly increased odds of myocardial injury (Table 3). The multivariable binary logistic regression model was statistically significant [*χ*^2^(7) = 349.340, *p* < 0.001], explained 43.3% (Nagelkerke *R*^2^) of the variance in myocardial injury and correctly classified 78.6% of cases. The model fit the data well using the Hosmer-Lemeshow goodness-of-fit test [*χ*^2^(8) = 12.485, *p* = 0.131].

**Figure 3:**
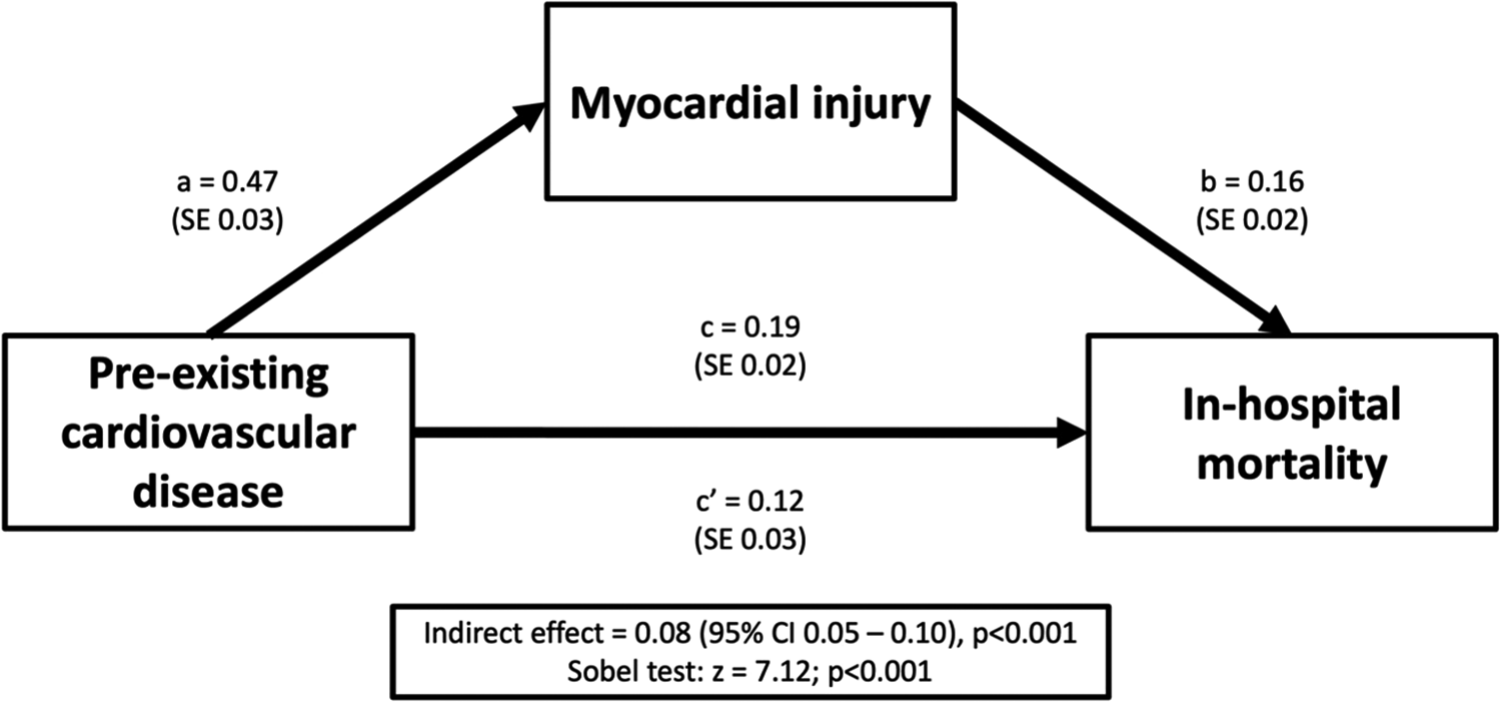
Mediation analysis diagram for pre-existing cardiovascular disease, myocardial injury and in-hospital mortality.

The mediation analysis diagram is depicted in Figure 3. Overall, pre-existing cardiovascular disease had a significant indirect effect on in-hospital mortality through myocardial injury (indirect effect = 0.08, 95% CI: 0.05–0.10; *p* < 0.001), and this mediation effect was confirmed by a significant Sobel test (*z* = 7.12, *p* < 0.001). These results suggest that myocardial injury partially mediates the relationship between pre-existing cardiovascular disease and in-hospital mortality.

## 4. Discussion

This prospective, multicentre observational study of 1,567 adult, hospitalised patients investigated the association between pre-existing cardiovascular disease and outcomes of mortality, clinical cardiovascular complications and myocardial injury. Pre-existing cardiovascular disease was frequently seen in hospitalised patients with COVID-19 and independently associated with significantly increased odds of mortality and myocardial injury, without significant difference in other clinical cardiovascular complications such as arrhythmias, cardiomyopathy or heart failure, pulmonary embolism and myocarditis. Apart from age, other cardiovascular risk factors such as diabetes, hypercholesterolemia and hypertension had no significant impact on mortality, clinical cardiovascular complications or myocardial injury. However, chronic kidney disease was independently associated with significantly increased odds of mortality and myocardial injury. Cumulatively, these findings suggest that pre-existing cardiovascular disease rather than cardiovascular risk factors portend increased mortality in hospitalised patients with COVID-19. This association is partially mediated by myocardial injury, rather than other clinical cardiovascular complications.

The incidence of pre-existing cardiovascular disease in our study was comparable to other international studies. However, data regarding the prognostic impact of pre-existing cardiovascular disease and other cardiovascular risk factors in these studies is conflicting (7, 10, 11). A multicentre, American study of 5,133 patients found a significant association between cardiovascular risk factors and mortality, rather than pre-existing cardiovascular disease. However, this study focused on patients admitted to the intensive care unit only in the early phase of the COVID-19 pandemic and limited the definition of pre-existing cardiovascular disease to coronary artery disease, heart failure and atrial fibrillation (10). A prospective cohort study of 1,604 patients from the Netherlands also found a significant association between cardiovascular risk factors such as hypertension, hypercholesterolemia and diabetes and mortality, however this analysis could not adjust for the presence of pre-existing cardiovascular disease as this information was not routinely collected and cardiovascular medication use was used as a surrogate exposure instead (12). A cohort study in the United Kingdom of 1,721 patients found comparable results to us, with pre-existing cardiovascular disease rather than cardiovascular risk factors associated with increased mortality (7). Moreover, patients with heart failure and particularly those with reduced ejection fraction have been identified as a high-risk cohort with poorer outcomes from COVID-19 (13).

The greater mortality risk associated with pre-existing cardiovascular disease, rather than other cardiovascular risk factors such as diabetes and hypertension, observed in our study lends to questions about the mechanisms that underpin this signal. A potential mechanism is that patients with pre-existing cardiovascular disease have an increased propensity for myocardial injury, which in combination with the extra-cardiac manifestations of COVID-19 due to a multisystem inflammatory response, lend to a poorer prognosis (14–16). This notion is supported by autopsy series in patients with COVID-19 demonstrating myocardial infiltration with macrophages and multiple thrombi in the coronary circulation; thereby suggesting diffuse myocardial inflammation and microvascular thrombi as the histopathological drivers of cardiac involvement in acute COVID-19 (17, 18). The significant mediation analysis finding in our study and the lack of a significant association between pre-existing cardiovascular disease and clinical cardiovascular complications, further reinforces the potential role of myocardial injury in mediating poorer outcomes in this population. Notably, most deaths in patients both with and without pre-existing cardiovascular disease in our study were not cardiovascular in nature. Given the predominance of other causes of mortality, the mediatory role of myocardial injury in all-cause, in-hospital mortality may reflect its role as a sensitive marker of systemic illness severity rather than a mechanistic pathway for mortality (19). Further studies examining the role of anti-inflammatory and immunomodulatory medications in mitigating myocardial injury, systemic illness severity and mortality in patients with pre-existing cardiovascular disease specifically are needed. Moreover, an increased burden of cardiovascular disease has been described in the longer term after COVID-19 infection, with putative mechanisms including a persistent, aberrantly hyperactive immune response (20, 21). The longer-term implications of myocardial injury during acute COVID-19 infection in patients with pre-existing cardiovascular disease remains to be explored.

There are some limitations to this analysis. Firstly, whilst we were able to adjust for potential confounding factors such as diabetes and age in this study, wamcthe AUS-COVID Registry lacks sufficient data on body mass index and does not include information on race, which are potentially significant confounders that are not accounted for in the multivariable models. Additionally, the registry only reports on in-hospital mortality, and long-term data is not available. Furthermore, there are limitations related to missing data, as the study relies on data extracted from electronic medical records during a pandemic setting without routine investigations, which may result in incomplete information, particularly when studying myocardial injury as an outcome. Moreover, information regarding adequate or inadequate treatment of cardiovascular risk factors such as diabetes, hypercholesterolemia and hypertension in patients included in this study are lacking and should be considered when interpreting our findings regarding the effect of these cardiovascular risk factors on patient outcomes. Despite these limitations, this study is significant as it utilizes the first and largest multicentre Australian registry that focuses on cardiovascular comorbidities among adult patients hospitalized with COVID-19. The Australian context for the COVID-19 pandemic remains uniquely relevant to current care for COVID-19 patients as it represents a healthcare system that was not overwhelmed by the COVID-19 pandemic, where hospital systems could maintain the necessitated high level of care to all patients with COVID-19. Another strength of our study is that it includes all hospitalized patients with laboratory-proven COVID-19, including patients admitted to the intensive care unit, lending to its increased generalisability to a wider population.

## 5. Conclusion

Pre-existing cardiovascular disease portends significantly higher mortality in patients hospitalised with COVID-19. This association may be partly explained by increased risk of myocardial injury among patients with pre-existing cardiovascular disease which in turn is a marker associated with higher mortality.

## Data Availability

The original contributions presented in the study are included in the article, further inquiries can be directed to the corresponding author.
